# Endogenous Amyloid-formed Ca^2+^-permeable Channels in Aged 3xTg AD Mice

**DOI:** 10.1093/function/zqad025

**Published:** 2023-05-26

**Authors:** Shuangtao Li, Xiaoyu Ji, Ming Gao, Bing Huang, Shuang Peng, Jie Wu

**Affiliations:** Brain Function and Disease Laboratory, Shantou University Medical College, Shantou, Guangdong 515041, China; Brain Function and Disease Laboratory, Shantou University Medical College, Shantou, Guangdong 515041, China; Department of Neurosurgery, First Affiliated Hospital, Shantou University Medical College, Shantou, Guangdong 515041, China; Department of Neurobiology, Barrow Neurological Institute and St. Joseph’s Hospital and Medical Center, Phoenix, AZ 85013, USA; Brain Function and Disease Laboratory, Shantou University Medical College, Shantou, Guangdong 515041, China; Department of Neurosurgery, First Affiliated Hospital, Shantou University Medical College, Shantou, Guangdong 515041, China; School of Sport and Health Sciences, Guangzhou Sport University, Guangzhou 510500, China; Key Laboratory of Sports Technique, Tactics and Physical Function of General Administration of Sport of China, Scientific Research Center, Guangzhou Sport University, Guangzhou 510500, China; Brain Function and Disease Laboratory, Shantou University Medical College, Shantou, Guangdong 515041, China; Department of Neurosurgery, First Affiliated Hospital, Shantou University Medical College, Shantou, Guangdong 515041, China; Department of Neurobiology, Barrow Neurological Institute and St. Joseph’s Hospital and Medical Center, Phoenix, AZ 85013, USA

## Abstract

Alzheimer’s disease (AD), the leading cause of dementia, is characterized by the accumulation of beta-amyloid peptides (Aβ). However, whether Aβ itself is a key toxic agent in AD pathogenesis and the precise mechanism of Aβ-elicited neurotoxicity are still debated. Emerging evidence demonstrates that the Aβ channel/pore hypothesis could explain Aβ toxicity, because Aβ oligomers are able to disrupt membranes and cause edge-conductivity pores that may disrupt cell Ca^2+^ homeostasis and drive neurotoxicity in AD. However, all available data to support this hypothesis have been collected from “in vitro” experiments using high concentrations of exogenous Aβ. It is still unknown whether Aβ channels can be formed by endogenous Aβ in AD animal models. Here, we report an unexpected finding of the spontaneous Ca^2+^ oscillations in aged 3xTg AD mice but not in age-matched wild-type mice. These spontaneous Ca^2+^ oscillations are sensitive to extracellular Ca^2+^, ZnCl_2_, and the Aβ channel blocker Anle138b, suggesting that these spontaneous Ca^2+^ oscillations in aged 3xTg AD mice are mediated by endogenous Aβ-formed channels.

## Graphical Abstract

**Graphical Abstract:**
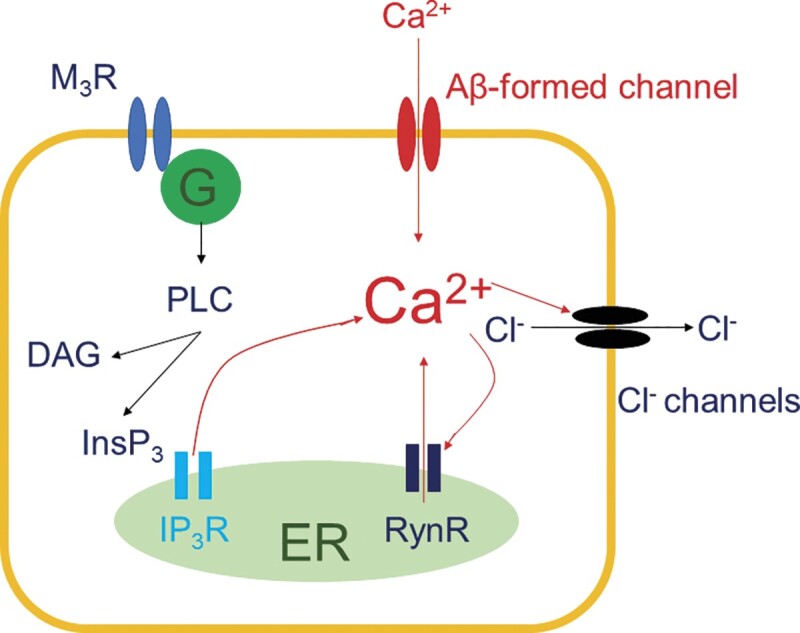

## Introduction

Alzheimer’s disease (AD), the leading cause of dementia, is characterized by the accumulation of beta-amyloid peptides (Aβ) in senile plaques in the brain of affected patients.^1^ Many cellular mechanisms are thought to play important roles in the development and progression of AD pathogenesis, but Aβ deposit-induced toxicity is still considered to be one of, if not the most, important factors in the pathogenesis of AD.^2^ Several lines of evidence support the Aβ channel/pore hypothesis stating that Aβ is able to disrupt membranes by causing pore formation.^3–6^ Therefore, Aβ toxicity can be explained at least in part on the basis of dysregulation of Ca^2+^ homeostasis by direct lipid disruption.^3,7,8^ This hypothesis was based on microscopic conductance changes induced by Aβ pore formation in artificial membranes that were highly complex and showed cation selectivity.^9,10^ Recent evidence shows that Aβ, similar to gramicidin, caused micro and macro perforation of cellular membranes, leading to neurotoxicity by a Ca^2+^-dependent mechanism in cultured neurons.^7,8^,^11–13^ The Aβ channel/pore shares several properties: hetero-dispersity, irreversibility, non-selectivity, long open times, blockade by zinc, inhibition by Anle138b, and enhancement by “aging” or acidic pH.^14^ These properties would lead to cells gradually becoming “leaky,” leading to loss of ionic gradients, dysregulation of calcium homeostasis, and high consumption of energy supplies. Although numerous studies have laid the foundation for this hypothesis, the predominant evidence is indirect and limited to in vitro system tests. Ideally, if one could record ionic currents under patch-clamp conditions on a cell, isolated from relevant regions in AD animal models, after all the native ion channels on such a hypothetical cell were blocked without altering the physiology, one could expect empirical evidence that would be direct, widely accepted and most useful to the understanding of AD.^15^ Based on this type of experimental setup, we provide direct evidence of an endogenous Aβ channel/pore in aged 3xTg AD mice.

The pancreatic acinar cell is a classical cell model, for studies of Ca^2+^ signal transduction mechanisms, because it has been possible to directly obtain considerable insight into intracellular Ca^2+^ handling under both normal and pathological conditions.^16^ Unlike nerve and endocrine, as well as muscle cells, exocrine cells such as pancreatic acinar cells are non-excitable cells and do not possess voltage-gated Ca^2+^ channels, and the cytosolic Ca^2+^ signals governing pancreatic acinar secretion are primarily generated by release of Ca^2+^ from intracellular stores, principally the endoplasmic reticulum (ER).^17–19^ For example, acetylcholine (ACh) can cause physiological cytosolic Ca^2+^ oscillatory signals (monitored by changes in the Ca^2+^-activated Cl^−^ current) through the G protein-IP_3_ pathway. Moreover, additional factors work together to keep the intracellular Ca^2+^ homeostasis in acinar cells, such as the plasma membrane Ca^2+^-activated ATPase, sarco(endo) plasmic reticulum Ca^2+^ activated ATPase and store-operated channels.

As a highly innervated organ, the pancreas shares a number of striking pathophysiologic similarities in Aβ deposition comparing to that in brain^20^. AD can cause the Aβ deposition in the pancreases of mice overexpressing human APP, suggesting that Aβ deposits may occur in organs other than the brain.^20^ Here, we show that Aβ deposits can be detected in pancreatic acinar cells in aged 3xTg AD model mice. Furthermore, unexpected spontaneous Ca^2+^ oscillations were found in such AD mouse acinar cells. Since mouse pancreatic acinar cells do not express voltage-gated Ca^2+^ channels, we hypothesize that the spontaneous Ca^2+^ oscillations mediated through endogenous Aβ-formed Ca^2+^-permeable channels in these aged AD mice.

## Materials and Methods

C57BL/6 J mice were purchased from Beijing Vital River Laboratory Animal Technology company. 3xTg AD mice, 12–24-mo-old, were a gift from Prof. Diling Chen (Guangdong Institute of Microbiology, China), who has published a paper using 3xTg AD mice.^21^ This AD mouse carries transgenes encoding mutants of presenilin-1 (PS1; M146V), amyloid precursor protein (APP; swe), and tau (P301L). These mutant genes lead to rapid accumulation of amyloid in mice and premature dysfunction of synaptic transmission and long-term potentiation.^22^ All mice were kept in groups under constant standard conditions of temperature and humidity, with *ad libitum* access to food and water, and on a 12-h light/dark cycle. All experimental procedures using mice conform to the Medical Animal Care and Welfare Committee and were approved by the Laboratory Animal Ethics Committee of Shantou University Medical College.

Single isolated mouse pancreatic cells were prepared as previously described.^23–26^ Briefly, wild-type or 3xTg AD mice were anesthetized with isoflurane. The pancreas was quickly removed and a small amount of collagenase solution was injected into the pancreas for digestion (150–200 U/mL, 18 min, 37°C; Wako Pure Chemicals, Osaka, Japan). At the end of the collagenase digestion, the cell suspension was gently triturated with a pipette to further dissociate the cells, and then the cells were washed several times with standard external solution (composition listed below). Thereafter, 200 µL aliquots of the suspension were transferred to a 35-mm culture dish containing 2 mL of standard external solution. Dissociated cells were typically used within 3 h after dissociation. All experiments were performed at 22 ± 1°C.

As previously reported,^23–26^ patch-clamp conventional whole-cell recordings were used to record Ca^2+^-activated Cl^−^ currents to monitor intracellular Ca^2+^ signaling oscillations. When the recording pipette was filled with K^+^-containing pipette solution, the resistance was 3–4 MΩ. After a GΩ seal was formed between the cell membrane and the pipette, the Aβ perforated whole-cell conformation was formed as judged by gradual (5–30 min) reduction of the access resistance (to <60 MΩ). Whole-cell recording mode was achieved by brief suction. The cells were held at −30 mV and the series resistance was not compensated. Transmembrane currents were recorded with a patch clamp amplifier (Axopatch 200B; Molecular Devices; Sunnyvale, CA, USA).

The standard extracellular solution contained (in m m): 140 NaCl, 4.7 KCl, 1.2 MgCl_2_, 1 CaCl_2_, 1.13 MgCl_2_, 10 glucose, and 10 HEPES, pH 7.3 adjusted using Tris-base. A Ca^2+^-free solution was prepared by replacing Ca^2+^ with Na^+^ (142 m m NaCl and 0 m m Ca^2+^) and adding 1 m m EGTA. The pipette solution for whole-cell recordings contained (in m m): 140 KCl, 0.24 EGTA, 1.13 MgCl_2_, 5 Na_2_ATP, 10 glucose, and 10 HEPES, pH 7.2. ACh, 2APB, and atropine sulfate used in this study were purchased from Sigma (St. Louis, MO, USA). Ruthenium red was purchased from Wako Chemical, and CdCl_2_ and ZnCl_2_ were purchased from Macklin (Shanghai Macklin Biochemical Co., Ltd). For external drug application, a “U-tube” rapid application system was employed. Data were filtered at 2 kHz, acquired at 5 kHz, and digitized online (Clampex 10.6 software, Digidata 1550B, Axon Instruments, Union City, CA, USA). All data were displayed and stored on a computer.

### Thioflavin S Staining

To identify the Aβ accumulation, the thioflavin S staining was used, samples were prepared as described.^27^ Briefly, 12-mo-old 3xTg mice or wild type (WT) mice of the same age were anesthetized, and the brain and pancreas were fixed by 4% paraformaldehyde perfusion through the heart. Paraffin-embedded blocks were prepared by sequential dehydration in graded ethanol before embedding, and tissues were serially sectioned to a thickness of 4 μm and mounted on pre-coated Poly-l-lysine glass slides. Sections were first pre-incubated with potassium permanganate solution and oxalic acid, then placed in 3% sodium borohydride solution for 5 min. Staining was performed using filtered 0.05% Thioflavin S (Sigma) in 50% ethanol for 30 min in the dark, and differentiated with two changes of 80% ethanol for 10 s. This was followed by 3 washes with large volumes of distilled water and an incubation step in 5× PBS buffer at 4°C for 30 min. Finally, slides were briefly rinsed in PBS and covered with coverslips using Vectashield Hard Set mounting media with DAPI (Vector). Slides were allowed to set in the dark at 4°C and imaged immediately thereafter. Fluorescence images were acquired on a Zeiss LSM800 confocal microscope.

### Preparation of oAβ_1-42_

For the oligomerization of Aβ_1–42_ (oAβ), a 1 m m Aβ solution was prepared by dissolving as-synthesized Aβ_1–42_ powder (DGpeptides Co.) in 1,1,1,3,3,3-hexafluoroisopropanol, and blow drying the liquid with nitrogen to form a peptide film at the bottom of the centrifuge tube. The Aβ_1–42_ peptide membrane was resuspended by adding DMSO and sonicated under sterile conditions for 10 min before use, then diluted to a 100 μm stock solution in PBS at 4°C. For the application of monomers, the 100 μm stork solution was directly diluted to the required concentration with standard extracellular solution. For the preparation of oligomers, the 100 μm stork solution was vortexed (15 s), centrifuged, and transferred to a 4°C freezer for 24 h. To avoid fibril formation, all samples were used within one day.

### Statistical Analysis

The net charge of the current was obtained by dividing the current area by the cell membrane capacitance (Cm) over a certain time (usually for 2 min). Before values were measured from the baseline of spontaneous Ca^2+^ oscillations from 3xTg AD mice for approximately 2 min (normalized to 1) and were compared to the changes induced by drugs exposure. Data are presented as mean ± SEM. One-way ANOVA was employed to compare the time to first calcium spike generation at different concentrations of oAβ. Differences with *P* < 0.05 were considered significant.

## Results

### Unexpected Spontaneous Ca^2+^ Oscillations in Aged 3xTg AD Mice

**Figure 1.**
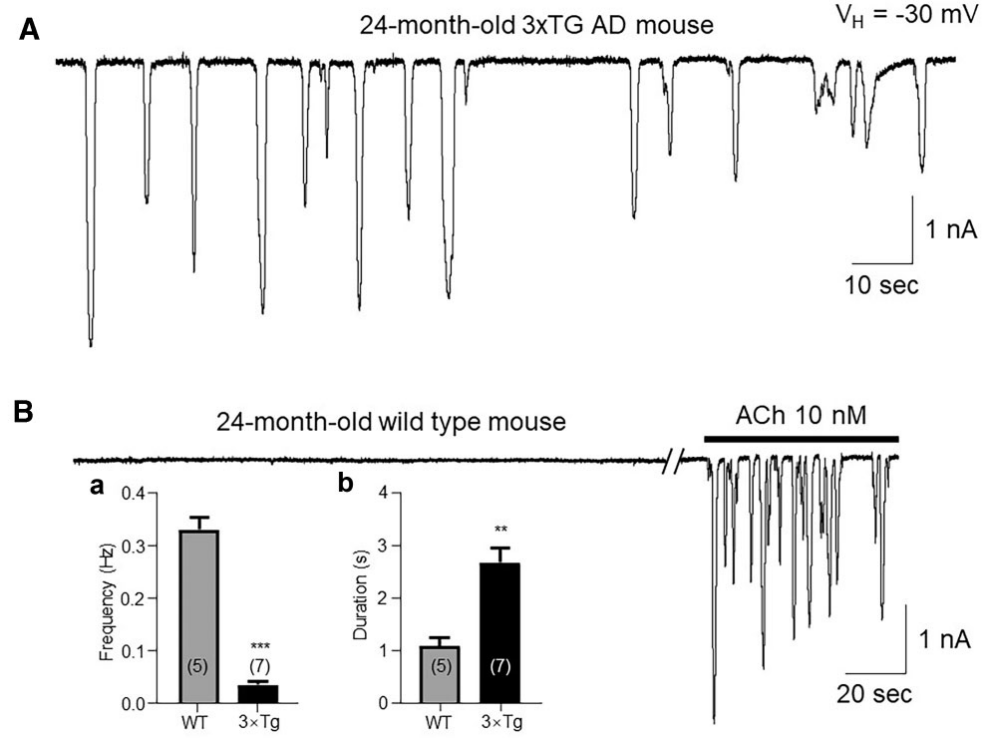
Representative traces show the spontaneous Ca^2+^ oscillations in freshly isolated pancreatic acinar cells from an aged 3xTg AD mouse (A), but not from an age-matched WT mouse (B). However, the WT cells showed oscillations induced by 10 n m Ach (B), suggesting that this cell exhibits good function. Comparing to ACh-induced oscillations in WT mice, the spontaneous Ca^2+^ oscillations of pancreatic acinar cells from an aged 3xTg AD mouse showed different frequency (1Ba) and duration (1Bb).

Usually, in mouse pancreatic acinar cells, Ca^2+^ oscillations can be induced by different stimulations, such as ACh, CCK, InsP3, or Ca^2+^ ion.^28^ However, by using patch-clamp whole-cell recordings in freshly dissociated pancreatic acinar cells from aged 3xTg AD mice, we found unexpected spontaneous Ca^2+^ oscillations (Figure 1A). In 16 aged (24-mo-old) 3xTg AD mice, we tested 34 acinar cells, and 23 (23/34) showed spontaneous Ca^2+^ oscillations, whereas in 8 age-matched WT mice, there were no detectable spontaneous Ca^2+^ oscillations in the 15 acinar cells tested (Figure 1B). In 5 WT cells (5/15), after whole-cell recording for 30 min (during which no responses were observed), 10 n m ACh was able to evoke Ca^2+^ oscillations (Figure 1B), suggesting that both cell function and recording quality are good. Compared to both frequency and individual spike duration of ACh (10 n m)-induced Ca^2+^ oscillation responses (Figure 1Ba, 0.29 ± 0.07 Hz, *n* = 5 cells, and Figure 1Bb, 1.08 ± 0.15 s, *n* = 5), the spontaneous Ca^2+^ oscillations exhibited much lower oscillational frequency but longer oscillatory spike duration (Figure 1Ba, 0.03 ± 0.02 Hz, *n* = 7 cells and Figure 1Bb, 2.67 ± 0.27 s, *n* = 7). Student’s *t*-test analysis showed that the difference in both oscillatory frequencies and duration between the spontaneous Ca^2+^ oscillations in pancreatic acinar cells from aged 3xTg AD mice and the ACh (10 n m)-induced Ca^2+^ oscillations in pancreatic acinar cells from age-matched WT mice were highly significant (*P* < 0.001).

### Spontaneous Ca^2+^ Oscillations Are Dependent on Extracellular Ca^2+^, and Sensitive to Zn^2+^ and Anle138b

**Figure 2.**
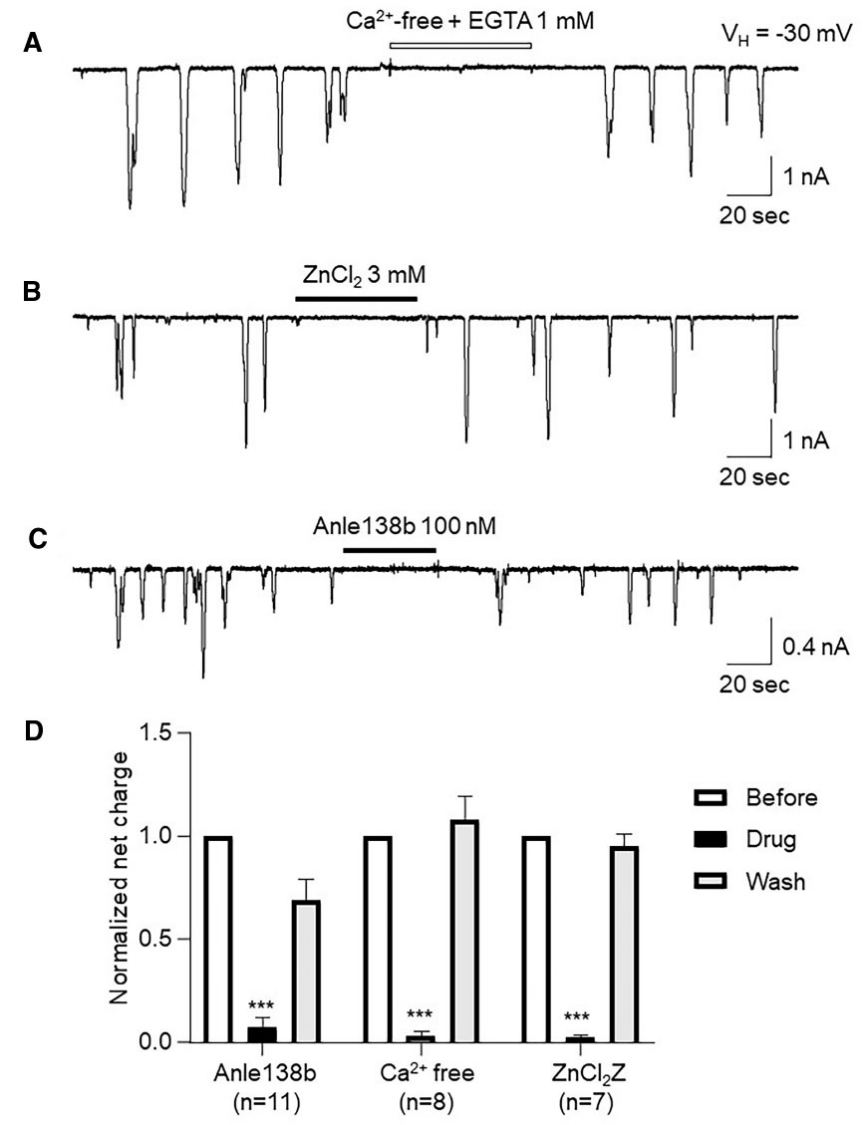
Representative traces showing spontaneous Ca^2+^ oscillations in pancreatic acinar cells from aged 3xTg AD mice are sensitive to the removal of extracellular Ca^2+^ (A), inhibition by 3 m m ZnCl_2_ (B), and inhibition by the Aβ channel blocker, Anle138b (C). Bar graph summarizes the presented data and shows that the 3 treatments-induced reduction of the spontaneous Ca^2+^ oscillations are highly significant (*P* < 0.0001) (D).

To explore the nature of these spontaneous Ca^2+^ oscillations, we performed 3 experiments. First, we removed extracellular Ca^2+^ by replacing standard extracellular solution with an extracellular Ca^2+^-free solution containing 1 m m EGTA. In 8 cells tested, we found that the removal of extracellular Ca^2+^ reversibly abolished these oscillations (Figure 2A), suggesting that the spontaneous Ca^2+^ oscillations are triggered by extracellular Ca^2+^ influx into cells. Then, we found that a high concentration of ZnCl_2_ (3 m m) eliminated spontaneous Ca^2+^ oscillations (Figure 2B, *n* = 7). Finally, we tested the effects of an amyloid (Aβ) channel blocker, Anle138b, and found that Anle138b (100 n m) completely inhibited the spontaneous Ca^2+^ oscillations (Figure 2C, *n* = 11), suggesting that the spontaneous Ca^2+^ oscillations in aged AD acinar cells are elicited by extracellular Ca^2+^ efflux into cell through the Aβ-formed channels. Statistical analysis (Figure 2D) demonstrates that all 3 treatments (removal of extracellular Ca^2+^, ZnCl_2_, and Anle138b) induced reduction of spontaneous Ca^2+^ oscillations are highly significant (*P* < 0.0001).

### Spontaneous Ca^2+^ Oscillations Are Mediated Through Ca^2+^-induced Ca^2+^ Release (CICR), Rather Than InsP_3_-induced Ca^2+^ Release (IICR)

**Figure 3.**
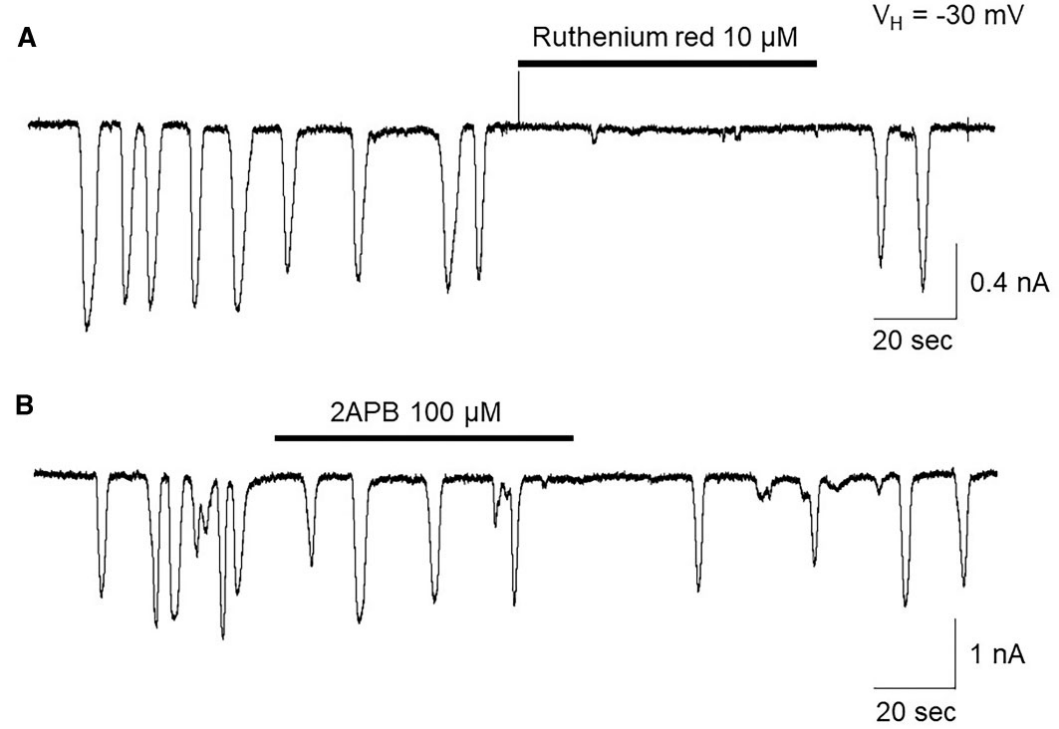
Representative traces showing that the spontaneous Ca^2+^ oscillations in pancreatic acinar cells from aged 3xTg AD mice are sensitive to ruthenium red (Ca^2+^-induced Ca^2+^-release blocker) (A), but not to 2APB (B) (store-operated Ca^2+^ entry blocker).

**Figure 4.**
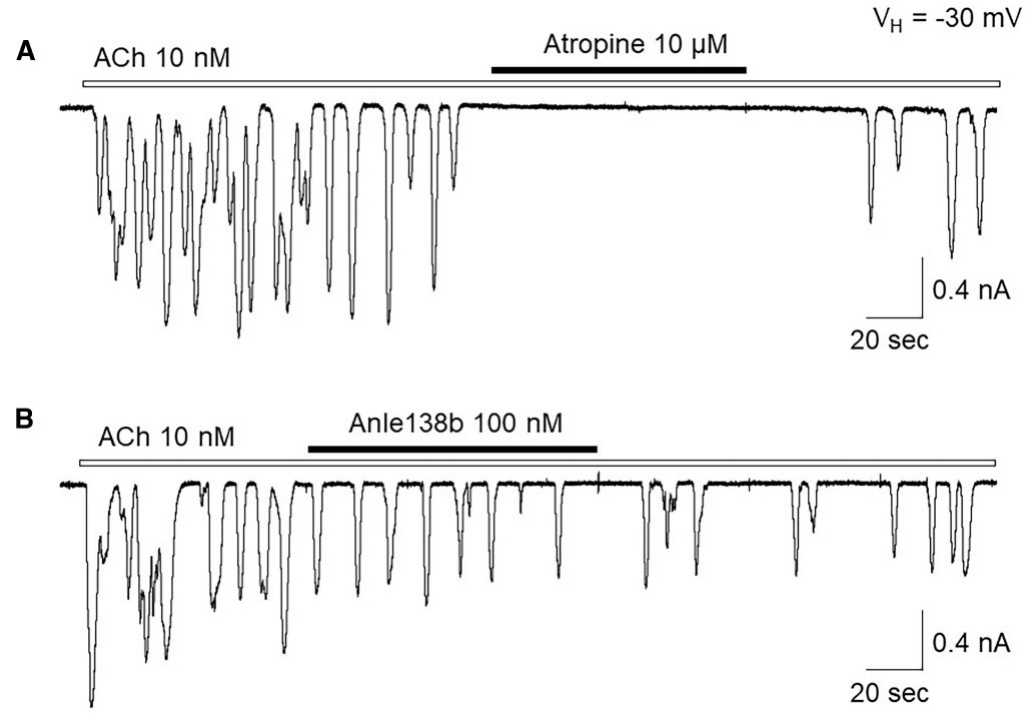
Representative traces showing that in aged acinar cells from 3xTg AD mice, ACh (10 n m)-induced Ca^2+^ oscillations can be blocked by atropine (A), but not by Anle138b (B), indicating that Anle138b is not able to block ACh (10 n m) induced the Ca^2+^ oscillations.

In these experiments, we further identified which intracellular Ca^2+^-signal pathways (CICR and/or IICR) mediated the spontaneous Ca^2+^ oscillations. As shown in Figure 3A, in 6 cells tested, the CICR blocker ruthenium red (10 μm) inhibited the spontaneous Ca^2+^ oscillations. To test whether the IICR participates in the spontaneous Ca^2+^ oscillations, we performed 2 experiments. First, we tested the effects of 2APB, both InsP_3_ receptor and store-operated Ca^2+^ channel blocker, and found that 100 μm 2APB failed to block the spontaneous Ca^2+^ oscillations (Figure 3B, *n* = 7). Second, we activated the IICR pathway, by application of ACh (10 n m), and showed that the ACh-induced Ca^2+^ oscillations can be blocked by 10 μm atropine (Figure 4A, *n* = 6), but cannot be blocked by 100 n m Anle138b (Figure 4B, *n* = 5). These results suggest that the spontaneous Ca^2+^ oscillations in acinar cells from aged 3xTg AD mice are mediated by extracellular Ca^2+^ influx into cytosol through the Aβ-formed channels, subsequently triggering the CICR signal pathway.

### Aβ-like Peptides Accumulate in the Pancreas of Aged 3xTg AD Mice

**Figure 5.**
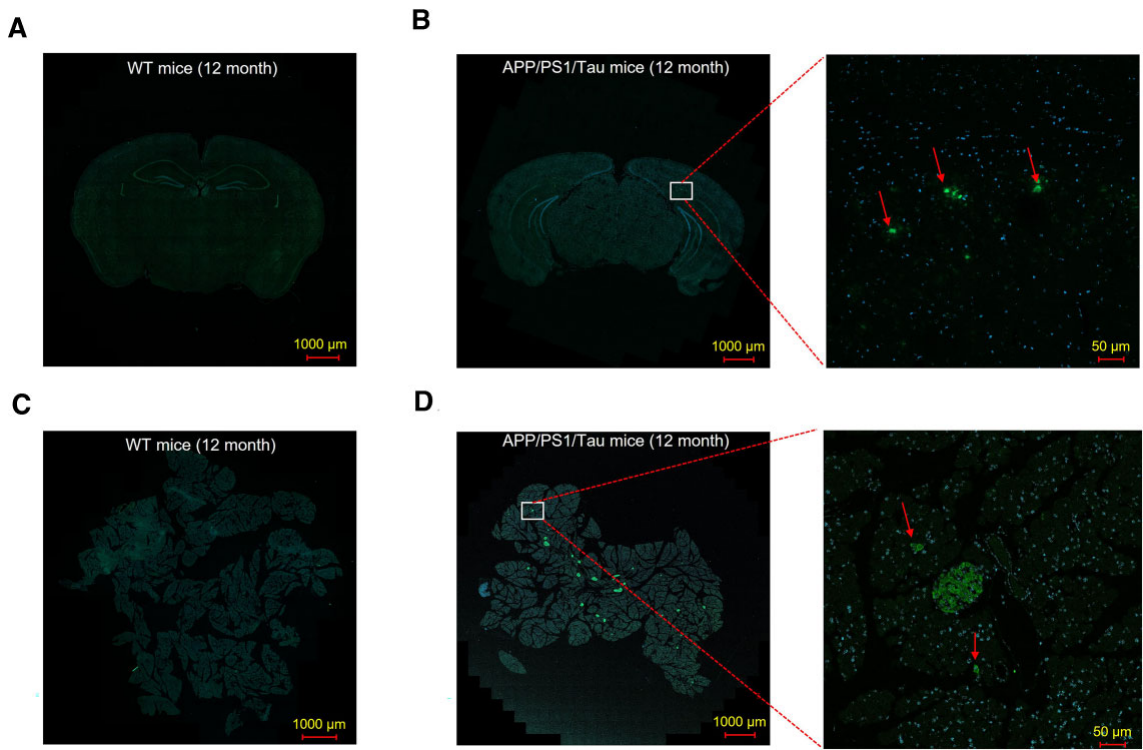
Thioflavin staining of Aβ deposition in the pancreas of 3xTg AD mice. Positive staining showing Aβ deposition in brain tissue (hippocampal section) of a 12-mo-old 3xTg AD mouse (B), but no staining of Aβ deposition in an aged-matched WT hippocampal section (A). Similarly, in pancreatic tissue sections, Aβ deposits were seen in an AD mouse (5D), but not in a WT mouse (5C).

It has been reported that APP is expressed in the normal mouse pancreas, and in the APP/PS1 mouse model of AD, APP is overexpressed within pancreatic islets.^29^ There are also extensive amyloid deposits in the pancreas of 8-mo-old APP/PS1 transgenic mice.^30^ In addition, transgenic mice overexpressing both amyloid beta-protein and perlecan in pancreatic acinar cells.^31,32^ We detected Aβ deposition, in the pancreas of 3xTg AD mice, using thioflavin staining. Brain tissue (hippocampal sections) of 3xTg AD mice was used as a positive control. Hippocampal sections of 12-mo-old 3xTg AD mice showed large numbers of Aβ deposits (Figure 5B), which were not found in age-matched hippocampal sections from WT mice (Figure 5A). In pancreatic tissue sections, similar Aβ deposits were observed in 12-mo-old 3xTg AD mice (Figure 5D), but were not seen in WT mice (Figure 5C).

### Exogenous Aβ Induces Spontaneous Ca^2+^ Oscillations in Pancreatic Acinar Cells From Adult Mice

**Figure 6.**
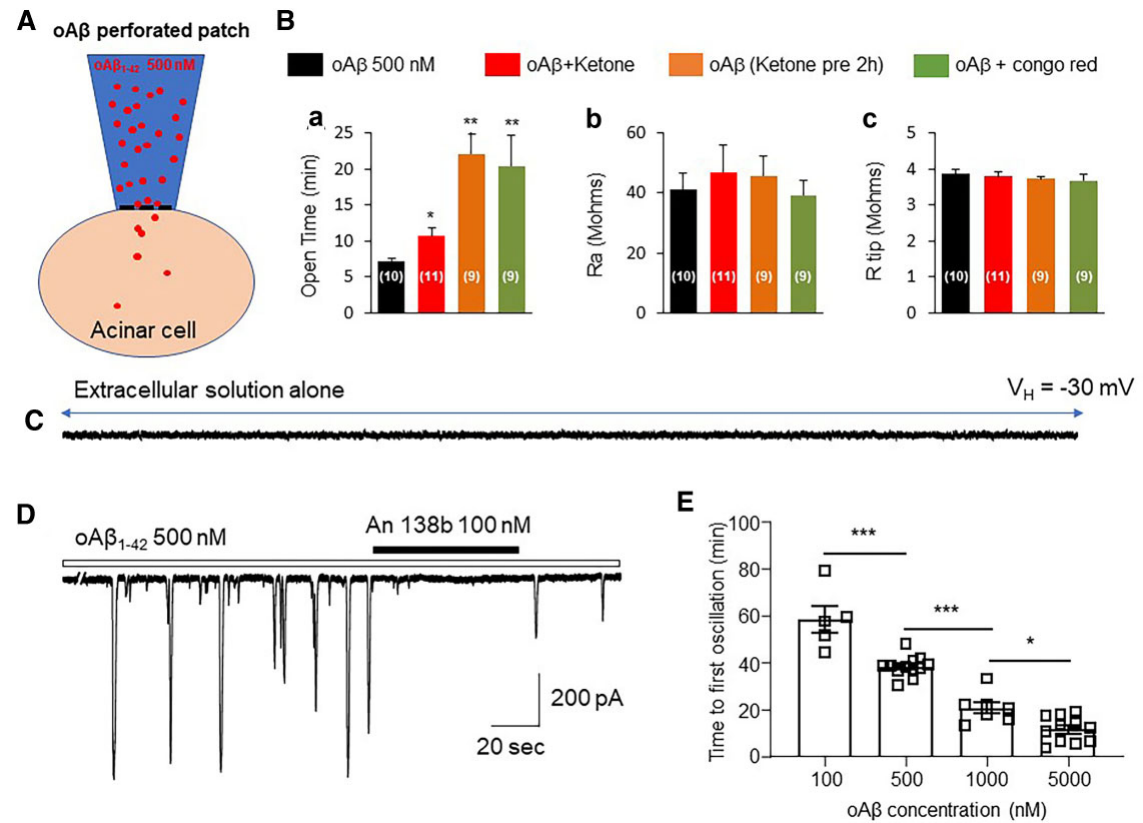
Exogenous oAβ channel formation in adult (6-mo-old) mice. (A) Cartoon showing the oAβ channels. (B) The oAβ channel activity under different conditions: 500 n m oAβ in pipette solution alone; 500 n m oAβ+10 μm ketone in pipette solution; 500 n m oAβ in pipette solution after pretreatment of cells with 10 μm ketone for 2 h; and 500 n m oAβ and 10 μm Congo red in pipette solution. (Ba) Time course of oAβ-perforated channels. (Bb) Series resistance (Ra) of oAβ-formed channels. (Bc) Electrode resistance during oAβ channel formation. (C) Patch-clamp whole-cell recording under voltage-clamp mode showed no detectable current when perfused with normal standard extracellular solution. (D) Representative trace of Ca^2+^ oscillations induced by bath-perfusion of 500 n m oAβ for about 30 min, and abolishment by Anle138b. (E) Bar graph showing dependence of oAβ-induced oscillations on bath-perfused oAβ concentration.

Data presented thus far indicate that pancreatic acinar cells of aged 3xTg AD mice display spontaneous Ca^2+^ oscillations induced by extracellular Ca^2+^ influx into cells through the Aβ channels likely formed by endogenous Aβ. To further confirm this, we mimicked Aβ channel formation by addition of exogenous oAβ to pancreatic acinar cells, which have no spontaneous Ca^2+^ oscillations, from 6-mo-old WT mice (Figure 6C). First, we tested the ability of oAβ (500 n m) to induce Ca^+^ oscillations by using perforated patch recording (Figure 6A). After establishment of a “giga seal” for about 5–30 min, the membrane series resistance gradually decreased to about 40 MΩ, indicating formation of a perforated whole-cell recording configuration (Figure 6 Ba, left column). Based on our previous report, ketone prevents oAβ cell entry in cultured hippocampal neurons.^33^ Therefore, we examined the effects of 1 m m ketone (pretreatment for 2 h or added simultaneously with oAβ) on the time required for oAβ channel formation. Either ketone co-application (oAβ and ketone were added into peptide solution together) or ketone pre-treatment (treatment of cells with ketone first for 2 h, then do patch recording with electrode that oAβ and ketone were added into peptide solution together) significantly prolonged the formation of oAβ channels, based on perforated whole-cell configuration measurements (Figure 6Ba). Furthermore, we examined the effects of 10 μm congo red on the time of oAβ channel formation in the perforated whole-cell configuration, and found similar prolongation of oAβ channel formation (Figure 6Ba). Neither ketone nor congo red affected the recording conditions, including the series resistance (Figure 6Bb) and electrode tip resistance (Figure 6Bc). Then, we tested whether bath-perfusion of 500 n m oAβ could induce Ca^2+^ oscillations like the spontaneous oscillations that we observed in aged APP mice. Figure 6C shows that under whole-cell path-clamp recording conditions, dissociated adult acinar cells did not show any spontaneous Ca^2+^ oscillations before perfusion of oAβ (*n* = 4), but after bath perfusion of 500 n m oAβ for 30 min, Ca^2+^ oscillations occurred (Figure 6D, *n* = 10) in a time and oAβ concentration-dependent manners (Figure 6E). Importantly, the oAβ-induced Ca^2+^ oscillations could be blocked by the Aβ channel blocker, Anle138b (Figure 6D, *n* = 8).

### Pharmacological Properties of Exogenous Aβ-induced Ca^2+^ Oscillations in Pancreatic Acinar Cells From Adult Mice

**Figure 7.**
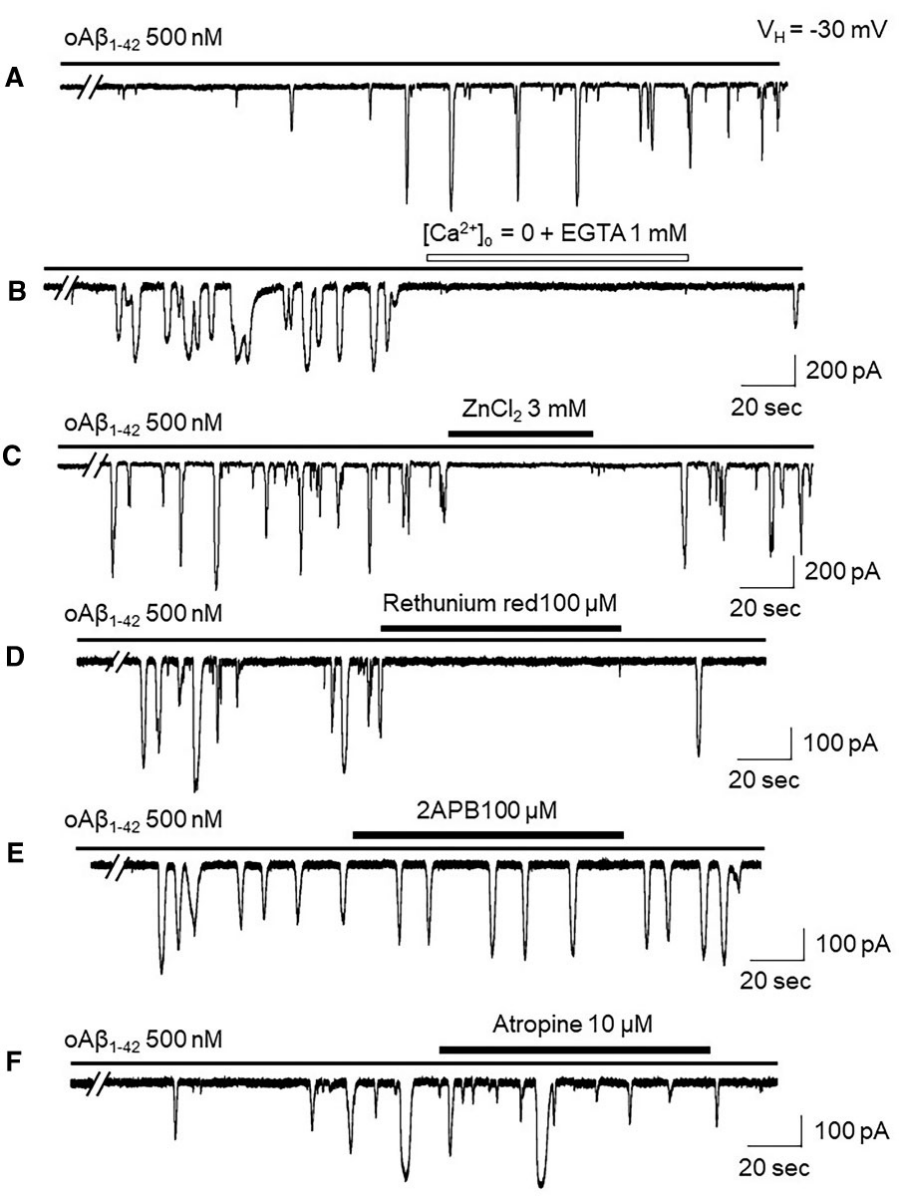
Pharmacological properties of exogenous Aβ-induced Ca^2+^ oscillations in pancreatic acinar cells from adult mice. Bath-perfusion of 500 n m oAβ induced Ca^2+^ oscillations (A), which were stopped by removal of extracellular Ca^2+^ (B), 500 n m oAβ-induced Ca^2+^ oscillations were inhibited by 3 m m ZnCl_2_ (C), and 100 μm ruthenium red (D), but not by either 100 μm 2APB (E) or 10 μm atropine (F).

To evaluate the nature of the exogenous Aβ-induced Ca^2+^ oscillations, we performed similar pharmacological tests as for the spontaneous Ca^2+^ oscillations found from the aged 3xTg AD mice (Figure 2). As shown in Figure 7, bath-perfusion of exogenous oAβ (500 n m) induced Ca^2+^ oscillations (Figure 7A, *n* = 6), and the removal of extracellular Ca^2+^ (by Ca^2+^-free extracellular solution containing 1 m m EGTA) reversibly stopped the oscillations (Figure 7B, *n* = 8). Like spontaneous Ca^2+^ oscillations found in the pancreatic acinar cells from the aged 3XTg AD mice, the Aβ-induced Ca^2+^ oscillations were sensitive to 3 m m ZnCl_2_ (Figure 1C, *n* = 6) and 100 μm ruthenium red (Figure 7D, *n* = 6), but were not sensitive to 100 μm 2APB (Figure 7E, *n* = 6). In 6 cells tested, we found that bath-applied atropine did not affect the oAβ-induced Ca^2+^ oscillations (Figure 7F, *n* = 4). These results suggest that the oAβ-induced Ca^2+^ oscillations are mediated through the Aβ channels.

### Comparison of Exogenous Aβ-induced Ca^2+^ Oscillations and ACh-induced Ca^2+^ Oscillations

**Figure 8.**
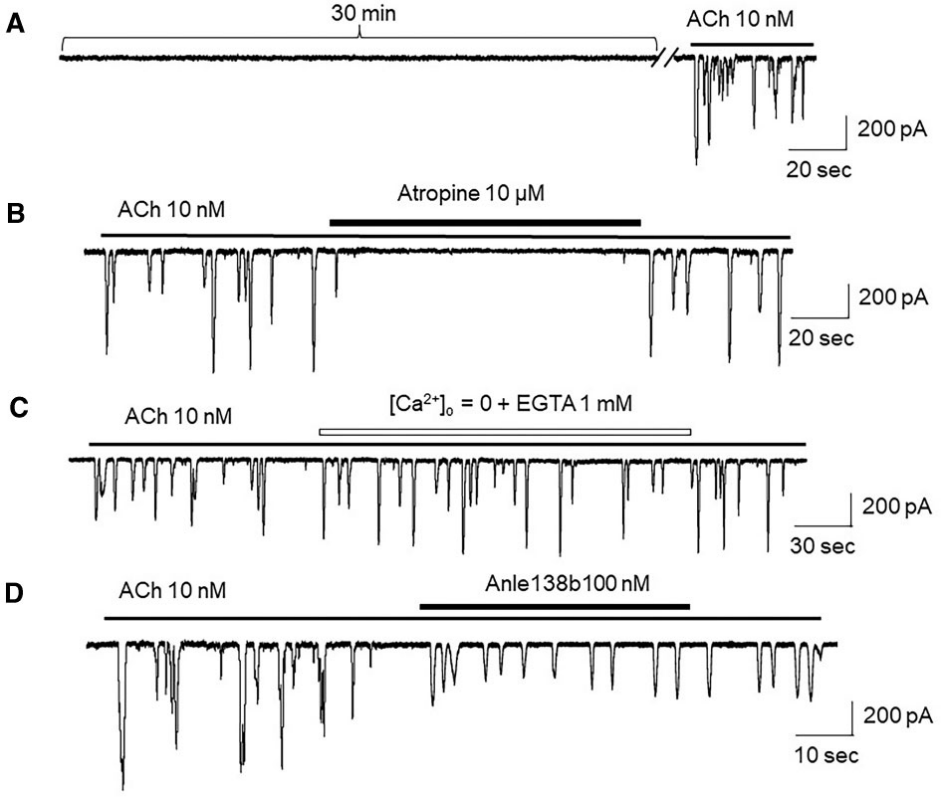
Pharmacological properties of exogenous ACh-induced Ca^2+^ oscillations in pancreatic acinar cells from adult mice. (A) Perfusion of standard extracellular solution did not induce current responses until bath-application of ACh. The ACh-induced Ca^2+^ oscillations were sensitive to atropine (B), but not to removal of extracellular Ca^2+^ (C) or treatment with Anle138b (D), VH = −30 mV.

In these experiments, we compared the oAβ-induced Ca^2+^ oscillations (see Figure 7) and the ACh-induced oscillations. Figure 8 shows that in acinar cells, from 6-mo-old WT mice, there were no detected oscillations during the 30 min recording, but bath application of ACh (10 n m) could induce typical Ca^2+^ oscillations (Figure 8A, *n* = 6). The ACh-induced Ca^2+^ oscillations were sensitive to the muscarinic receptor blocker, atropine (Figure 8B, *n* = 4), but were not sensitive to either removal of extracellular Ca^2+^ (Figure 8B, *n* = 6) or treatment with the Aβ channel blocker Anle138b (Figure 8C, *n* = 5). These results suggest that the trigger and pharmacology of ACh-induced Ca^2+^ oscillations are different from oAβ-induced Ca^2+^ oscillations.

## Discussion

The major and novel finding in this study is the unexpected spontaneous Ca^2+^ oscillations in aged 3xTg AD mice but not in age-matched WT mice. These spontaneous Ca^2+^ oscillations are sensitive to extracellular Ca^2+^ and high concentrations of ZnCl_2_, and more importantly can be eliminated by Anle138b, an Aβ-formed channel blocker, suggesting that these spontaneous Ca^2+^ oscillations in aged 3xTg AD mice are mediated by Ca^2+^ influx through endogenous Aβ-formed channels. To confirm this, we mimicked Aβ-formed channels in freshly isolated pancreatic acinar cells from adult (6-mo-old) WT mice, and used patch-clamp recording to show that oAβ in the recording electrode (500 n m) can perforate the membrane and form a pore, and bath-perfusion of exogenous 500 n m oAβcan induce Ca^2+^ oscillations that exhibit properties similar to the spontaneous Ca^2+^ oscillations observed in aged 3xTg AD mice. To our knowledge, this is the first evidence of the Aβ-formed channels by endogenous Aβ in aged 3xTg AD mice.

AD is a dementing, neurodegenerative disorder characterized by increased accumulation of Aβ, selective degeneration of forebrain cholinergic neurons, and progressive deficits in learning and memory. It has been postulated that Aβ triggers cytotoxicity that in turn causes AD, but the mechanisms involved remain elusive. The Aβ channel hypothesis has been postulated to explain Aβ toxicity, and states that Aβ is able to allosterically assemble into an ion channel structure embedded in a membrane lipid bilayer, resulting in membrane leakage and unbalanced Ca^2+^ homeostasis, leading to neuronal damage and death.^9^ In 1992, Hardy and Higgins reported that Aβ can eliminate neuronal calcium homeostasis, thus making neurons more vulnerable to environmental damage.^34^ In 1993, Arispe et al. used electrophysiological techniques to show that Aβ1–40 can combine with the planar lipid bilayer to further form a cation-selective channel, thereby demonstrating the ability of Aβ to form channels and in turn induce an imbalance of Ca^2+^ homeostasis, which may underlie Aβ neurotoxicity.^35^ Although this hypothesis sounds very interesting and is likely an important mechanism of Aβ toxicity, the evidence supporting this hypothesis was collected using artificial membranes or cell culture at a nascent stage.^3,9,10^,^36–40^ There are 2 major concerns for this hypothesis: (1) Aβ concentrations used to form the channels are too high and the lack of the evidence demonstrating such channels can be formed by endogenous Aβ,^15^ and (2) the existence of Aβ channels has not been shown in AD animal models. These knowledge gaps significantly diminish enthusiasm for this hypothesis. In this study, we unexpectedly found the spontaneous Ca^2+^ oscillations in pancreatic acinar cells isolated from aged 3xTg AD AD model mice, but not from age-matched WT mice. This had surprised us because there are no classical voltage-gated Ca^2+^ channels expressed in pancreatic acinar cells. Then, the question was how do these Ca^2+^ ions enter the cell? Considering that the pancreas shares a number of striking pathophysiologic similarities in Aβ deposition compared to that in brain, and AD also can cause Aβ deposition in the mouse pancreas upon overexpression of human APP, suggesting that Aβ deposits may occur in other organs than the brain.^20^ Therefore, we hypothesized that Aβ channels may mediate these spontaneous Ca^2+^ oscillations in aged 3xTg AD mice. We designed 4 experiments to test this hypothesis. First, we excluded the possibility that the spontaneous Ca^2+^ oscillations were mediated through classical ACh receptor—G-protein—IP_3_–Ca_2+_ (IICR) pathway by showing that atropine had no effect on the spontaneous oscillations (Figure 1C). Then, we identified that the spontaneous Ca^2+^ oscillations are mediated by extracellular Ca^2+^ ion influx into cytosol, because the oscillations were quickly limited by removal of extracellular Ca^2+^, and are triggered by CICR because the ryanodine receptor antagonist ruthenium red was able to eliminate the oscillations (Figure 1D). More importantly, the Aβ channel blocker Anle138b completely blocked the oscillations (Figure 1E). These results suggest that in aged 3xTg AD mice, there are Aβ depositions in the pancreas (Figure 5D),^29–32^ where Aβ forms Ca^2+^-permeable Aβ channels and causes spontaneous Ca^2+^ oscillations. To further confirm this, we mimicked Aβ-formed channels in pancreatic acinar cells, dissociated from 6-mo-old adult mice, by bath-perfusion of oAβ. Under whole-cell path-clamp recording conditions, these adult acinar cells did not show any spontaneous Ca^2+^ oscillations before perfusion of oAβ (Figure 2A), but after a 30-min bath perfusion of 500 n m oAβ, Ca^2+^ oscillations occurred (Figure 2B). The time course of oAβ-induced Ca^2+^ oscillations depended on the oAβ concentration (Figure 2C). Importantly, the oAβ-induced Ca^2+^ oscillations exhibited the similar features as the spontaneous Ca^2+^ oscillations of acinar cells from aged 3xTg AD mice, including the dependence of extracellular Ca^2+^ (Figure 2D), sensitivity to ruthenium red (Figure 2E), and inhibition by Anle138b (Figure 2F).

To our knowledge, this is the first evidence that the Aβ formed channels from endogenous oAβ in an aged AD model. Aβ accumulates in pancreatic tissues and pancreatic acinar cells are not expressed in classical voltage-gated Ca^2+^ channels, which gives us the ability to find the special phenomenon of spontaneous Ca^2+^ oscillations. Although pancreatic acinar cells possess very selective Ca^2+^ release activated Ca^2+^ (CRAC) channels, and these are vitally important for continuous Ca^2+^ signaling as the intracellular (ER) store is not infinite. Direct electrophysiological recordings of the CRAC current in mouse pancreatic acinar cells have been reported,^41^ in which, it is shown directly that the CRAC current is acutely (and reversibly) blocked by 100 µm 2APB, the exact same concentration that is shown in Figure 4B, in which, 2APB did not block the spontaneous Ca^2+^ oscillations in the 3xTg AD mice, strongly suggesting that the spontaneous Ca^2+^ oscillations are not mediated through the CRAC channels.

The pancreatic acinar cell is a classical model for studies of Ca^2+^ signal transduction mechanisms because it enables one to directly obtain considerable insight into intracellular Ca^2+^ handling under both normal and pathological conditions.^42^ Unlike nerve and endocrine cells, as well as muscle cells, exocrine cells such as pancreatic acinar cells are non-excitable and do not possess voltage-gated Ca^2+^ channels, and the cytosolic Ca^2+^ signals governing pancreatic acinar secretion are primarily generated by the release of Ca^2+^ from intracellular stores, principally the ER^17, 19, 42^ (Figure 1). For example, ACh can cause physiological cytosolic Ca^2+^ oscillatory signals through a G protein-IP_3_ pathway (Figure 1). The increase in intracellular Ca^2+^ generates Ca^2+^-induced Ca^2+^ release by affecting the open-state probability of the IP_3_R or RyR channels.^43^ As a highly innervated organ, pancreas shares a number of pathophysiologic similarities to Aβ deposition in the brain .^20^ Our results show that Aβ deposits can be detected in both pancreatic acinar cells and hippocampal neurons in aged APP AD model mice (Figure 5). Based on this finding, we expect that Aβ channels are also expressed in brain neurons, such as hippocampal and cortical neurons. Therefore, Aβ toxicity can be explained in part on the basis of dysregulation of Ca^2+^ homeostasis by Aβ channels.^3,7,8^ Recent evidence shows that Aβ, similar to gramicidin, causes micro and macro perforation in cellular membranes to induce neurotoxicity by a Ca^2+^-dependent mechanism in cultured neurons.^7,8^,^11–13^ Aβ channels/pores share common properties of heterodispersity, irreversibility, non-selectivity, long open times, blockade by zinc, inhibition by congo red, and enhancement by “aging” or acidic pH.^14^ These properties lead to cell leakage, decreases in ionic gradients, dysregulation of calcium, and consumption of energy supplies.

We show that Aβ deposits in pancreatic acinar cells can form Ca^2+^ permeable channels/pores in aged 3xTg AD mice. Notably, we demonstrate the ability of Aβ to perforate intact animal cell membranes, supporting the Aβ channel/pore hypothesis. Our results also show that Aβ-mediated disruption of Ca^2+^ homeostasis in acinar cells increases intracellular Ca^2+^ concentrations, which may be involved in Aβ toxicity. Recently, the failures of clinical trials associated with reducing Aβ deposition in mild AD patients raises the question of whether Aβ is the critical target for AD pathogenesis and treatment. Our findings suggest that Aβ may play an important role in triggering cell pathogenesis in the middle or late stages of AD. However, our results also suggest that pharmacological blockade of Aβ channels is a likely novel potential therapeutic strategy to improve AD pathogenesis and learning and memory deficits. Collectively, we identify an endogenous Aβ channel in an aged AD mouse model, providing new insight into the understanding of AD pathogenesis, and the intervention of AD pathological processes, as well as providing a potential treatment to improve AD cognitive deficits by targeting the Aβ channels.

### Limitations of the Study

In this study, we unexpectedly found spontaneous Ca^2+^ oscillations in pancreatic acinar cells isolated from aged 3xTg AD mice but not in age-matched WT mice. Since pancreatic acinar cells have no classical voltage-gated Ca^2+^ channels, which gives us a chance to find the endogenous Aβ-formed channels in aged 3xTg AD mice. On the other hand, this cell preparation limits our finding directly linking Aβ-formed channels and cognitive deficits in AD model mice. We believe that it is hard to find endogenous Aβ-formed channels in neurons because neurons express various types of voltage-gated Ca^2+^ channels, whereas pancreatic acinar cells have no such Ca^2+^ channels. To overcome this limitation, we need to extend our study to hippocampal neurons and evaluate the effects of Aβ-formed Ca^2+^ channel blockers (eg, Anle138b) on Aβ-induced neuronal toxicity and neurodegeneration, and also on cognitive deficits and learning and memory behavioral impairment in AD models. These experiments are ongoing.

## Data Availability

The datasets generated during the current study are available from the corresponding author on reasonable request.
